# Kidney Parameters with Tirzepatide in Obesity with or without Type 2 Diabetes

**DOI:** 10.1681/ASN.0000000764

**Published:** 2025-06-13

**Authors:** Hiddo J.L. Heerspink, Allon N. Friedman, Petter Bjornstad, Daniel H. van Raalte, David Cherney, Dachuang Cao, Luis-Emilio Garcia-Pérez, Adam Stefanski, Ibrahim Turfanda, Mathijs C. Bunck, Imane Benabbad, Ryan Griffin, Carolina Piras de Oliveira

**Affiliations:** 1 Department of Clinical Pharmacology and Pharmacy, University of Groningen, University Medical Center Groningen, Groningen, The Netherlands; 2 Indiana University School of Medicine, Indianapolis, Indiana; 3 University of Washington School of Medicine, Seattle, Washington; 4 Amsterdam University Medical Center, Amsterdam, The Netherlands; 5 Department of Medicine, Toronto General Hospital Research Institute, University Health Network, Toronto, Ontario, Canada; 6 Eli Lilly and Company, Indianapolis, Indiana

**Keywords:** albuminuria, chronic diabetic complications, CKD, creatinine, diabetes, diabetes mellitus, GFR, kidney, obesity

## Abstract

**Key Points:**

- People with obesity and/or type 2 diabetes are at higher risk of progressive kidney function loss.
- We assessed the association of tirzepatide use with kidney function parameters in people with overweight/obesity with or without type 2 diabetes.
- Tirzepatide treatment was associated with urine albumin-to-creatinine reduction after 24 weeks, which was sustained through week 72, and no change in eGFR.

**Background:**

Tirzepatide, a once-weekly, glucose-dependent insulinotropic polypeptide and glucagon-like peptide-1 receptor agonist, showed kidney-protective effects in people with type 2 diabetes at high cardiovascular disease risk. In this *post hoc* analysis of the SURMOUNT-1 and SURMOUNT-2 trials, we assessed the association of tirzepatide use with kidney function parameters in people with overweight/obesity with or without type 2 diabetes.

**Methods:**

In SURMOUNT-1, participants with overweight or obesity without type 2 diabetes were randomized to tirzepatide 5, 10, and 15 mg or placebo. In SURMOUNT-2, participants with type 2 diabetes were randomized to tirzepatide 10 and 15 mg or placebo. For this analysis, all tirzepatide groups were pooled in each trial. Assessments included change from baseline to week 72 for urine albumin-to-creatinine ratio (UACR) and eGFR. eGFR was assessed using creatinine-based eGFR, cystatin-C–based eGFR, and creatinine-cystatin-C–based eGFR (Cr-Cys-C-eGFR).

**Results:**

In SURMOUNT-1 (*N*=2539) and SURMOUNT-2 (*N*=938), the median (25th–75th percentile) baseline UACR was 6.0 (4.0–11.0) mg/g and 13.0 (6.0–35.1) mg/g, respectively. UACR estimated difference for tirzepatide versus placebo, at week 72 was−8.4% (95% confidence interval [CI], −14.7 to −1.6) for SURMOUNT-1 and −31.1% (95% CI, −40.9 to −19.7) for SURMOUNT-2. The UACR change was more pronounced among participants with baseline UACR ≥30 mg/g with placebo-corrected changes from baseline at week 72 of −42.3% (95% CI, −60.8 to −15.0) in SURMOUNT-1 and −55.2% (95% CI, −68.5 to −36.4) in SURMOUNT-2, respectively. In SURMOUNT-1, tirzepatide was associated with increased eGFR based on cystatin-C–based eGFR or Cr-Cys-C-eGFR estimation equations, with mean differences versus placebo at week 72 of 3.2 ml/min per 1.73 m^2^ (95% CI, 2.1 to 4.3) and 1.9 ml/min per 1.73 m^2^ (95% CI, 0.9 to 2.9), respectively. In SURMOUNT-2 at week 72, increases in both tirzepatide and placebo groups were observed for Cys-C or Cr-Cys-C-eGFR, with no between-group differences.

**Conclusions:**

In participants with obesity/overweight with or without type 2 diabetes, tirzepatide was associated with reduced albuminuria without adverse changes in eGFR.

**Clinical Trial registry name and registration number:**

SURMOUNT-1: NCT04184622; SURMOUNT-2: NCT04657003.

## Visual Abstract

**Figure:**
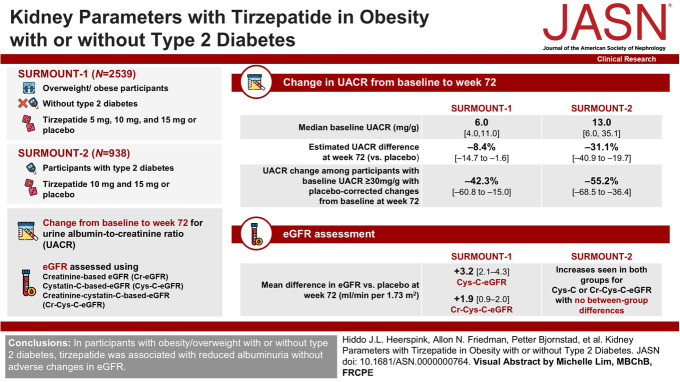

## Introduction

The prevalence of obesity has doubled since the early 90s, with over 2.5 billion people living with obesity or overweight in 2022.^1^ The prevalence of CKD has also risen, with over 800 million people affected worldwide in 2017.^2^ As obesity has consistently been identified as an independent risk factor for the development of CKD, it also often leads to comorbidities including hypertension or type 2 diabetes. These in turn are associated with higher risk of microvascular and macrovascular complications including CKD.^3,4^ Obesity can also directly contribute to CKD development through non–diabetes-related mechanisms.^5^ Current guidelines recommend weight management for people with CKD and excess weight.^6^ For people with type 2 diabetes, pharmacologic treatment options include renin-angiotensin-system (RAS) inhibitors, sodium-glucose cotransporter-2 inhibitors (SGLT2i), and the nonsteroidal mineralocorticoid receptor antagonist finerenone.^6^ Although these therapies are effective in slowing the progressive loss of kidney function, their effects on body weight are minimal, especially for people with established CKD.^7,8^ The introduction of novel therapies that simultaneously reduce body weight and offer kidney protection represents a potentially significant advancement in the therapeutic landscape.

Incretins, such as the glucose-dependent insulinotropic polypeptide (GIP) and glucagon-like peptide-1 (GLP-1), stimulate insulin and suppress glucagon secretion, thereby influencing key physiologic aspects of metabolism and homeostasis.^9^ The GLP-1 receptor agonists (RAs) have demonstrated reduction in major adverse cardiovascular events in people with or without type 2 diabetes and improved kidney outcomes in people with type 2 diabetes.^10–12^ Tirzepatide is a GIP and GLP-1 RA approved for the treatment of type 2 diabetes^13^ and obesity^14^ and has been shown to promote insulin secretion after meals,^15^ improve glycemic control, and reduce body weight.^16–18^ These effects of tirzepatide are more pronounced compared with GLP-1 RAs alone.^19^ In people with type 2 diabetes at high cardiovascular risk, 104 weeks of treatment with tirzepatide was associated with reduced albuminuria and a slower decline in eGFR compared with treatment with insulin glargine.^3^ Tirzepatide also markedly reduced body weight and improved glycemic control in people with overweight or obesity without or with type 2 diabetes in the SURMOUNT-1 and SURMOUNT-2 clinical trials.^16,17^ The effects of tirzepatide on albuminuria and eGFR in people with obesity or overweight without or with type 2 diabetes are unknown. We therefore performed a *post hoc* analysis of the SURMOUNT-1 and SURMOUNT-2 trials to test the hypothesis that treatment with tirzepatide is associated with improvements in kidney parameters in these two participant populations.

## Methods

### Study Designs

This *post hoc* analysis included participants from the phase 3, double-blind, placebo-controlled, multicenter SURMOUNT-1 (NCT04184622; December 2, 2019) and SURMOUNT-2 (NCT04657003; December 1, 2020) trials. The full study designs and primary results have been previously reported,^16,17^ and the trial designs are summarized in Supplemental Table 1.

In brief, in SURMOUNT-1, participants were randomized 1:1:1:1 to once-weekly, subcutaneous tirzepatide (5, 10, or 15 mg) or placebo for 72 weeks, followed by a 4-week safety follow-up period without treatment. In SURMOUNT-2, participants were randomized 1:1:1 to once-weekly, subcutaneous tirzepatide (10 or 15 mg) or placebo for 72 weeks, followed by a 4-week safety follow-up period without treatment. For both trials, treatment was an adjunct to lifestyle intervention (dietary counseling and at least 150 minutes per week of physical activity). Both trials were conducted according to the International Conference on Harmonisation Good Clinical Practice guidelines and the Declaration of Helsinki. All participants provided written informed consent.

### Participants

Both trials included adult participants with obesity or overweight and ≥1 obesity-related comorbidity (Supplemental Table 1). In SURMOUNT-1, participants with type 2 diabetes were excluded, whereas in SURMOUNT-2, participants were required to have type 2 diabetes. The full eligibility criteria have previously been reported^16,17^ and are presented in brief in Supplemental Table 1.

### Outcomes

This *post hoc* analysis assessed the outcomes of change from baseline to week 72 for urine albumin-to-creatinine ratio (UACR) and eGFR. UACR-related outcomes included mean percent change from baseline in UACR in the pooled tirzepatide population, the proportions of participants with normal albuminuria (UACR <30 mg/g), microalbuminuria (UACR ≥30 to ≤300 mg/g), and macroalbuminuria (UACR >300 mg/g) at 72 weeks of follow-up, and the proportions of participants whose UACR decreased by at least 30% from baseline.

eGFR was assessed using creatinine-based eGFR (Cr-eGFR) using the CKD Epidemiology Collaboration creatinine equation 2021, cystatin-C–based eGFR (Cys-C-eGFR), and creatinine-cystatin-C–based eGFR (Cr-Cys-C-eGFR) using the CKD Epidemiology Collaboration creatinine-cystatin equation 2021. Change in eGFR was assessed in participants with baseline Cr-Cys-C-eGFR <60 and ≥60 ml/min per 1.73 m^2^. Safety outcomes included adverse events related to kidney function, electrolyte balance, and fractures as reported by investigators.

### Statistical Analysis

Analyses were conducted on the efficacy analysis set, which included randomized participants excluding off-treatment data. For SURMOUNT-1, the tirzepatide 5, 10, and 15 mg groups were pooled for all analyses, whereas for SURMOUNT-2, the tirzepatide 10 and 15 mg groups were pooled for all analyses. This approach was consistent with previous tirzepatide studies in the SURPASS program.^3,20^ We also analyzed the effects of tirzepatide for each dose group separately. The changes from baseline to week 72 in UACR and eGFR were assessed using a mixed model for repeated measures with on-treatment data, which included the following variables: baseline value, stratification factors (country, sex, prediabetes for SURMOUNT-1, or antihyperglycemic medication used at randomization for SURMOUNT-2), treatment, time (categorical), and treatment×time (type 3 sum of squares). An unstructured covariance matrix was used to allow for general patterns of SDs and correlations across the repeated outcome measurements. The proportion of participants with normal albuminuria at week 72 was estimated using logistic regression with missing data at week 72 imputed by the last observed carried forward.

Pearson correlations between changes in body weight and changes in Cr-eGFR were calculated and presented in scatterplots. In this exploratory *post hoc* analysis, 95% confidence intervals (CI) were presented to facilitate the interpretation of the findings.

All analyses were performed using SAS software version 9.4 or above (SAS Institute Inc., Cary, NC).

## Results

### Participant Disposition and Baseline Characteristics

In the SURMOUNT-1 trial, 2539 participants were randomly assigned to receive tirzepatide (5, 10, or 15 mg; *N*=1896) or placebo (*N*=643). In the SURMOUNT-2 trial, 938 participants were randomized to tirzepatide 10 or 15 mg (*N*=623) or placebo (*N*=315). Overall, 86% and 87% of participants in the SURMOUNT-1 and SURMOUNT-2 trials, respectively, completed the study treatment period on study drug.

Baseline characteristics were well balanced between the tirzepatide and placebo groups in both trials (Table 1). The median UACR (25th–75th percentile) in SURMOUNT-1 and SURMOUNT-2 was 6.0 (4.0–11.0) and 13.0 (6.0–35.0) mg/g, respectively. The mean (SD) Cr-eGFR and Cys-C-eGFR were 100 (17) and 96 (19) ml/min per 1.73 m^2^ in SURMOUNT-1, respectively. The corresponding Cr-eGFR and Cys-C-eGFR values in SURMOUNT-2 were 97 (17) and 81 (21) ml/min per 1.73 m^2^, respectively (Table 2).

**Table 1:** Baseline demographics and clinical characteristics

Characteristic	SURMOUNT-1					SURMOUNT-2			
	PBO (*N*=643)	TZP 5 mg (*N*=630)	TZP 10 mg (*N*=636)	TZP 15 mg (*N*=630)	Total (*N*=2539)	PBO (*N*=315)	TZP 10 mg (*N*=312)	TZP 15 mg (*N*=311)	Total (*N*=938)
Age, yr	44 (13)	46 (13)	45 (12)	45 (12)	45 (13)	55 (11)	54 (11)	54 (11)	54 (11)
Female, *n* (%)	436 (68)	426 (68)	427 (67)	425 (68)	1714 (68)	159 (50)	158 (51)	159 (51)	476 (51)
Duration of obesity, yr	14.0 (10.7)	14.0 (10.8)	14.7 (11.1)	14.8 (10.8)	14.4 (10.8)	18.1 (11.7)	17.6 (12.0)	17.5 (11.0)	17.7 (11.5)
Body weight, kg	105 (21)	103 (21)	106 (23)	106 (23)	105 (22)	102 (22)	101 (21)	100 (20)	101 (21)
BMI, kg/m^2^	38.2 (6.9)	37.4 (6.6)	38.2 (7.0)	38.1 (6.7)	38.0 (6.8)	36.6 (7.3)	36.0 (6.4)	35.7 (6.1)	36.1 (6.6)
Systolic BP, mm Hg	123 (13)	124 (12)	124 (13)	123 (13)	123 (13)	131 (12)	131 (12)	130 (12)	131 (12)
Diastolic BP, mm Hg	80 (8)	79 (8)	80 (8)	79 (8)	80 (8)	79 (8)	80 (8)	80 (9)	80 (8)
Duration of diabetes, yr	—	—	—	—	—	8.8 (6.2)	8.8 (6.9)	8.0 (6.4)	8.5 (6.5)
HbA1c, %	5.6 (0.4)	5.6 (0.4)	5.6 (0.4)	5.6 (0.4)	5.6 (0.4)	8.0 (0.8)	8.0 (0.8)	8.1 (1.0)	8.0 (0.9)
**Concomitant medications, *n* (%)^a^**									
SGLT2i	—	—	—	—	—	67 (21)	64 (21)	62 (20)	193 (21)
ARB	89 (14)	92 (15)	85 (13)	110 (18)	376 (15)	109 (35)	104 (33)	104 (33)	317 (34)
ACEI	57 (9)	58 (9)	49 (8)	40 (6)	204 (8)	78 (25)	74 (24)	72 (23)	224 (24)

Data are mean (SD) unless stated otherwise. ACEI, angiotensin-converting enzyme inhibitor; ARB, angiotensin II receptor blocker; BMI, body mass index; HbA1c, glycated hemoglobin; PBO, placebo; SGLT2i, sodium-glucose cotransporter-2 inhibitor; TZP, tirzepatide.

a Includes all medications for each subgroup listed under the Preferred Terms within the Anatomical Therapeutic Chemical level 4 code.

**Table 2:** Baseline kidney function

Characteristic	SURMOUNT-1			SURMOUNT-2		
	Placebo (*N*=643)	Pooled Tirzepatide 5, 10, and 15 mg (*N*=1896)	Total (*N*=2539)	Placebo (*N*=315)	Pooled Tirzepatide 10 and 15 mg (*N*=623)	Total (*N*=938)
Cr-eGFR, ml/min per 1.73 m^2^, mean (SD)	99.8 (17.5)	99.9 (17.2)	99.9 (17.3)	96.2 (18.2)	98.2 (17.1)	97.5 (17.5)
Cys-C-eGFR, ml/min per 1.73 m^2^, mean (SD)	96.0 (18.6)	95.4 (19.3)	95.5 (19.1)	80.4 (22.1)	81.9 (20.8)	81.4 (21.2)
Cr-Cys-C-eGFR, ml/min per 1.73 m^2^, mean (SD)	100.4 (17.0)	100.2 (17.4)	100.2 (17.3)	90.3 (20.6)	92.1 (19.1)	91.5 (19.7)
**Cr-Cys-C-eGFR category, *n* (%)**						
<60 ml/min per 1.73 m^2^	8 (1)	44 (2)	52 (2)	25 (8)	33 (5)	58 (6)
≥60 ml/min per 1.73 m^2^	635 (99)	1852 (98)	2487 (98)	289 (92)	590 (95)	879 (94)
UACR, mg/g, median (IQR)	6.0 (4.0–11.0)	6.0 (4.0–11.0)	6.0 (4.0–11.0)	13.0 (6.0–32.0)	13.0 (6.0–36.0)	13.0 (6.0–35.1)
**UACR category, *n* (%)**						
Normal albuminuria (UACR <30 mg/g)	589 (92)	1726 (91)	2315 (91)	228 (72)	435 (70)	663 (71)
Microalbuminuria (UACR ≥30 to ≤300 mg/g)	48 (8)	147 (8)	195 (8)	76 (24)	152 (25)	228 (24)
Macroalbuminuria (UACR >300 mg/g)	6 (0.9)	18 (1)	24 (0.9)	11 (4)	34 (6)	45 (5)

The denominator may vary based on the total *N* in each category. Cr-Cys-C-eGFR, creatinine-cystatin-C–based eGFR; Cr-eGFR, creatinine-based eGFR; Cys-C-eGFR, cystatin-C–based eGFR; IQR, interquartile ratio; UACR, urine albumin-to-creatinine ratio.

### Changes in UACR

**Figure 1:**
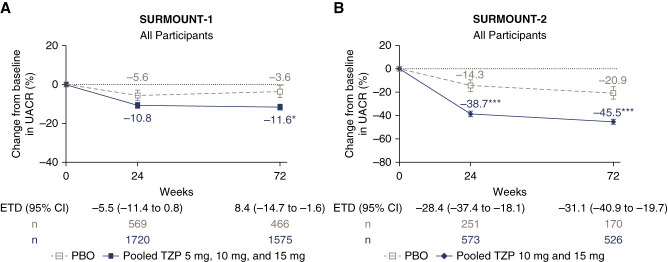
**Change from baseline in UACR (%) over 72 weeks of tirzepatide treatment for all participants.** (A) SURMOUNT-1 and (B) SURMOUNT-2. *P < 0.05, ***P < 0.001 versus placebo. Data are estimate (SEM). CI, confidence interval; ETD, estimated treatment difference; PBO, placebo; TZP, tirzepatide; UACR, urine albumin-to-creatinine ratio.

Figure 1 shows the UACR percent change from baseline to week 72 in SURMOUNT-1 (Figure 1A) and SURMOUNT-2 (Figure 1B). In the placebo group of the SURMOUNT-1 trial, UACR geometric mean (% coefficient of variation) at week 24 was 7.2 mg/g (118.5), corresponding to an estimate (SEM) change from baseline of −5.6% (2.7), and this change was sustained until week 72. In the tirzepatide group, UACR geometric mean (% coefficient of variation) at week 24 was 7.0 mg/g (97.0), corresponding to an estimate (SEM) change from baseline of −10.8% (1.5), and this change was also sustained until week 72. Accordingly, the mean percentage UACR difference compared with placebo at week 72 was −8.4% (95% CI, −14.7 to −1.6).

**Figure 2:**
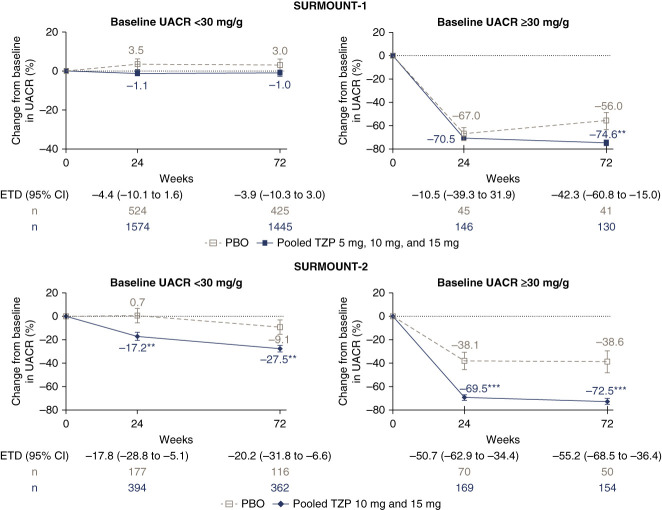
**Change from baseline in UACR (%) categories over 72 weeks of tirzepatide treatment in SURMOUNT-1 and SURMOUNT-2.** **P < 0.01, ***P < 0.001 versus PBO. Data are estimate (SEM).

In the placebo group of the SURMOUNT-2 trial, UACR estimate (SEM) change from baseline at week 24 was −14.3% (4.9) and further reduced to −20.9% (5.3) at week 72. In the tirzepatide group, the estimate (SEM) change from baseline in UACR was −38.7% (2.3) at week 24 and further reduced to −45.5% (2.2) at week 72. The mean difference in the change from baseline in UACR between the tirzepatide and placebo group at week 72 was −31.1% (95% CI, −40.9 to −19.7). In both trials, the reduction in UACR with tirzepatide compared with placebo was more pronounced among participants with baseline UACR ≥30 mg/g compared with those with baseline UACR <30 mg/g (Figure 2). Specifically, at week 72 in participants with UACR ≥30 mg/g in SURMOUNT-1, tirzepatide was associated with a relative reduction in UACR of −42.3% (95% CI, −60.8 to −15.0; *P* < 0.01) compared with placebo. In participants with UACR ≥30 mg/g in SURMOUNT-2, tirzepatide treatment was associated with a relative reduction in UACR of −55.2% (95% CI, −68.5 to −36.4; *P* < 0.001) compared with placebo.

**Figure 3:**
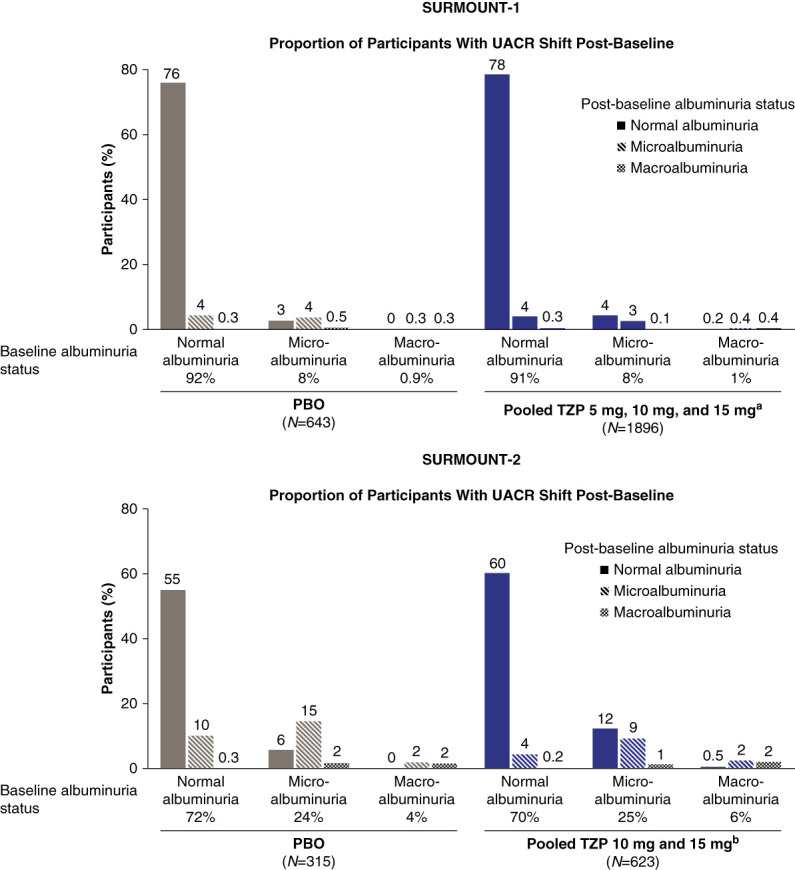
**Proportion of participants with UACR shift postbaseline in SURMOUNT-1 and SURMOUNT-2.** ^a^Baseline data were missing from four participants with normal albuminuria postbaseline in SURMOUNT-1. ^b^Baseline data were missing from two participants with normal albuminuria postbaseline in SURMOUNT-2.

In both SURMOUNT-1 and SURMOUNT-2, the proportion of participants with normal albuminuria at week 72 was higher in the tirzepatide groups (83% and 74%, respectively) compared with the placebo groups (79% and 61%, respectively; Figure 3). The proportion of participants in SURMOUNT-1 who experienced a ≥30% reduction in UACR from baseline at week 72 was higher in the tirzepatide group (34%) compared with the placebo group (28%). This was also true for participants in SURMOUNT-2 (58% for tirzepatide and 44% for placebo).

Subgroup analyses also demonstrated that the UACR change with tirzepatide compared with placebo was mostly consistent across various participant subgroups including those with baseline eGFR <90 or ≥90 ml/min per 1.73 m^2^ or subgroups of participants using or not using angiotensin-converting enzyme inhibitors/angiotensin II receptor blockers or SGLT2i (Supplemental Figures 1 and 2). The differences between tirzepatide doses and placebo in the UACR change from baseline at week 72 in SURMOUNT-1 and SURMOUNT-2 are presented in Supplemental Table 3. These data showed no evidence that the effects of tirzepatide on UACR were dose-dependent. Adjusting for concomitant changes in systolic BP showed similar change from baseline in UACR at week 72 with tirzepatide compared with placebo as in our main analysis, with a between group difference of −7.3% (95% CI, −13.7 to −0.4) in SURMOUNT-1 and −30.1% (95% CI, −40.1 to −18.5) in SURMOUNT-2. Analysis using multiple imputation instead also yielded similar results to our main analysis (data not shown).

### Changes in eGFR

**Figure 4:**
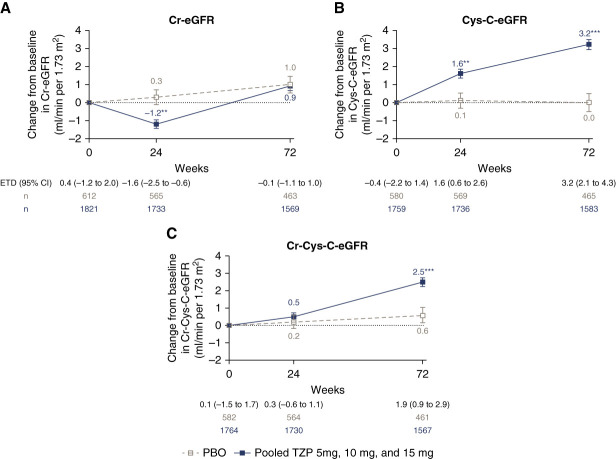
**Change from baseline in eGFR over 72 weeks of tirzepatide treatment for all participants in SURMOUNT-1.** (A) Cr-eGFR, (B) Cys-C-eGFR, and (C) Cr-Cys-C-eGFR. **P < 0.01, ***P < 0.001 versus placebo. Data are LSM (SEM). Cr-Cys-C-eGFR, creatinine-cystatin-C–based eGFR; Cr-eGFR, creatinine-based eGFR; Cys-C-eGFR, cystatin-C–based eGFR; LSM, least squares mean.

In SURMOUNT-1, the average change from baseline in Cr-eGFR was small and not statistically significant throughout the study. In the tirzepatide group, an initial decrease in Cr-eGFR was observed at week 24 (mean difference compared with placebo −1.6 ml/min per 1.73 m^2^). However, after week 24, eGFR values in the tirzepatide group returned to baseline and no eGFR difference between the tirzepatide and placebo groups was observed at week 72 (mean difference, −0.1 ml/min per 1.73 m^2^; 95% CI, −1.1 to 1.0). When the association of tirzepatide with eGFR was assessed using either Cys-C-eGFR or Cr-Cys-C-eGFR estimation equations, eGFR increased with mean differences compared with placebo at week 72 of 3.2 ml/min per 1.73 m^2^ (95% CI, 2.1 to 4.3) and 1.9 ml/min per 1.73 m^2^ (95% CI, 0.9 to 2.9), respectively (Figure 4 and Supplemental Table 4).

**Figure 5:**
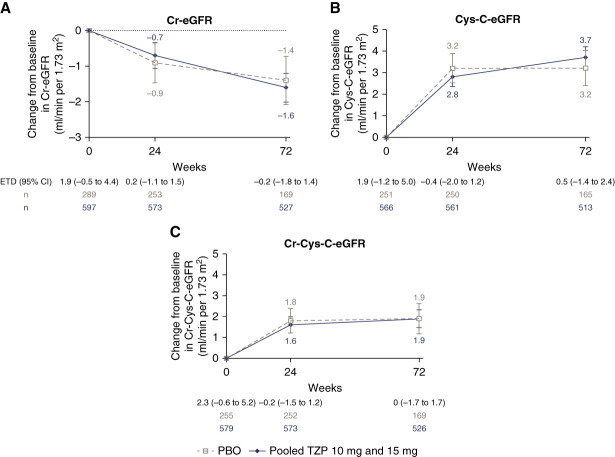
**Change from baseline in eGFR over 72 weeks of tirzepatide treatment for all participants in SURMOUNT-2.** (A) Cr-eGFR, (B) Cys-C-eGFR, and (C) Cr-Cys-C-eGFR. Data are LSM (SEM).

In SURMOUNT-2, Cr-eGFR declined over time in the placebo group. In the tirzepatide group, an initial decrease in eGFR (least squares mean [SEM]) of −0.7 (0.37) ml/min per 1.73 m^2^ was observed at week 24. Thereafter, eGFR remained stable throughout the 72-week follow-up. At week 72, the mean difference in the tirzepatide group compared with placebo group was −0.2 ml/min per 1.73 m^2^ (95% CI, −1.8 to 1.4). As for changes in Cys-C- or Cr-Cys-C-eGFR, increases in both the tirzepatide and placebo groups were observed with a small between-group difference for Cys-C-eGFR of 0.5 (95% CI, −1.4 to 2.4) and no between-group difference for Cr-Cys-C-eGFR throughout the 72-week follow-up period (Figure 5 and Supplemental Table 4).

Subgroup analyses demonstrated that the association of tirzepatide compared with placebo with eGFR changes was consistent in participants with baseline eGFR <60 or ≥60 ml/min per 1.73 m^2^ or baseline UACR <30 or ≥30 mg/g (Supplemental Figures 1 and 2).

### Correlations between Changes in Body Weight and eGFR

As published previously, all tirzepatide doses reduced body weight during the 72-week treatment period compared with placebo in SURMOUNT-1 and SURMOUNT-2.^16,17^ In SURMOUNT-1, there was no correlation in the placebo and tirzepatide groups between change from baseline in body weight and Cr-eGFR (all Pearson correlation coefficients <0.05; all *P* values > 0.05; Supplemental Figure 3). By contrast, in the placebo and tirzepatide groups, we observed modest, albeit statistically significant, correlations between changes in body weight and Cys-C-eGFR or Cr-Cys-C-eGFR (all Pearson correlation coefficients >0.09; Supplemental Figure 3). In SURMOUNT-2, there were no correlations between changes in body weight and eGFR during tirzepatide treatment regardless of whether eGFR was estimated with creatinine, cystatin C, or both (all Pearson correlation coefficients <0.05; Supplemental Figure 4). There were also no statistically significant correlations between these parameters in the placebo group.

### Safety

The overall safety profiles for both SURMOUNT-1 and SURMOUNT-2 have been previously reported.^16,17^ For kidney-related adverse events, renal and urinary disorders were reported by 2%–3% of participants across the tirzepatide groups in SURMOUNT-1, compared with 3% in the placebo group (Supplemental Table 2). AKI was reported by 0.3%–0.6% of participants across the tirzepatide groups, compared with 0.2% of participants in the placebo group. Increased blood potassium was reported only in the tirzepatide 5 mg group by two (0.3%) participants and none in the other tirzepatide groups or placebo group. One or more fractures were reported by 19 (1.0%) participants across the tirzepatide groups and by eight (1%) participants in the placebo group. In SURMOUNT-2, renal and urinary disorders were reported by 3%–5% of participants across the tirzepatide groups, compared with 4% in the placebo group. AKI was reported by 1% of participants treated with tirzepatide 10 mg compared with 0.3% in the placebo group. Increased blood potassium and decreased blood calcium were only reported in the tirzepatide 10 mg group by one (0.3%) participant. One or more fractures were reported by eight (1%) participants across the tirzepatide groups and by four (1%) participants in the placebo group.

## Discussion

In this *post hoc* analysis of two randomized placebo-controlled clinical trials in participants who had overweight or obesity without or with type 2 diabetes, tirzepatide was associated with a reduction in albuminuria, particularly among participants with elevated albuminuria at the start of the study. This finding supports and extends previous studies with GLP-1 RAs in participants with type 2 diabetes to a dual GIP/GLP-1 RA and to participants with overweight or obesity without type 2 diabetes. In this study, the analysis of changes in Cr-eGFR over time in participants treated with tirzepatide showed an initial dip followed by a reversal to baseline in participants in SURMOUNT-1, whereas in SURMOUNT-2, Cr-eGFR stabilized after an initial reduction. When eGFR was estimated with cystatin-C, eGFR increased over time in the tirzepatide group compared with the placebo group in participants in SURMOUNT-1, whereas in participants in SURMOUNT-2, Cys-C-eGFR increased in both the tirzepatide and placebo groups. Overall, tirzepatide was generally well tolerated with no imbalance between the tirzepatide and placebo groups in kidney-related adverse events.

In the overall population, the change in UACR with tirzepatide was more pronounced in participants with diabetes. Among participants with UACR ≥30 mg/g, clinically meaningful reductions in UACR of over 30% were observed with no clear evidence that these changes varied between participants with or without type 2 diabetes considering the overlapping CIs. The larger overall reduction in albuminuria in the diabetes (SURMOUNT-2) compared with the nondiabetes (SURMOUNT-1) trial may be explained by the larger proportion of participants with UACR ≥30 mg/g in SURMOUNT-2. The albuminuria reduction with tirzepatide was observed irrespective of RAS inhibitor or SGLT2i use, which are current guideline-recommended treatments to slow the progression of CKD.^21,22^ These data suggest that albuminuria reduction with tirzepatide is likely independent and additive to the proven kidney-protective effects of RAS inhibitors and SGLT2i. These results are relevant in the context of the FLOW trial that reported a 24% reduction in CKD progression and lower risk of death from cardiovascular-related or kidney-related causes (composite outcome) with the GLP-1 RA semaglutide compared with placebo treatment on top of RAS inhibition.^12^ In the FLOW trial, albuminuria was also reduced with semaglutide use. A *post hoc* analysis of the SURPASS-2 trial suggested additional albuminuria reduction with tirzepatide compared with semaglutide.^23^ Whether combined GIP and GLP-1 receptor activation with tirzepatide reduces albuminuria and eGFR decline more than GLP-1 receptor activation alone, and potentially improves kidney outcomes, requires a dedicated head-to-head kidney outcome trial.

Most participants in SURMOUNT-1 and SURMOUNT-2 had normal or modest albuminuria at baseline. Although the percentage reduction in albuminuria with tirzepatide in these participants was the same as in participants with higher degrees of albuminuria, the absolute reduction in albuminuria was less because of their lower starting levels. Large observational studies have demonstrated strong associations between the degree of albuminuria and the risk of kidney and cardiovascular outcomes, with no lower threshold below which this association attenuates.^24,25^ Observational studies have also documented strong associations between the percentage change in albuminuria over time and the risk of kidney outcomes, with consistent findings in subgroups of participants with no or modest albuminuria and more severe albuminuria.^26,27^ Collectively, these data imply that changes in albuminuria are associated with the same relative risk of CKD-related clinical outcomes regardless of albuminuria levels. Therefore, reductions in albuminuria observed in our analysis may be of clinical relevance also for people with obesity or overweight with modest albuminuria.

The reduced albuminuria with tirzepatide probably involves direct and indirect effects mediated through improvements in glycemic and body weight control. A mediation analysis of the SURPASS program in participants with type 2 diabetes indicated that approximately half of the albuminuria-lowering effect associated with tirzepatide compared with placebo or insulin treatment was explained by reductions in glycated hemoglobin and body weight.^23^ In addition, tirzepatide reduces fat accumulation and restores adipocytokine regulation, which may translate into reduced glomerular albumin filtration.^28–30^ Adiponectin is an insulin-sensitizing peptide that plays a role in lipid and glucose metabolism.^28,29^ In experimental models, it has been shown to restore the glomerular endothelial glycocalyx and reduced albumin permeability.^29^ Clinical studies have also suggested that tirzepatide may increase adiponectin.^28^ Other potential direct effects of tirzepatide that could ameliorate albuminuria and preserve kidney function may involve anti-inflammatory properties. It has been hypothesized that increased filtration of albumin causes excessive tubular reabsorption, resulting in inflammation.^31^ Tubular dysfunction may cause albuminuria due to decreased tubular albumin reabsorption.^31^ Experimental studies have also suggested that downregulating renin-aldosterone-axis activation, reducing lipotoxicity, immunomodulatory effects, and improving insulin sensitivity may be implicated in the kidney-protective effects of GLP-1 RAs.^5,32^ Mechanistic aspects of tirzepatide on kidney function are assessed in more detail in the ongoing prospective TREASURE-CKD (NCT05536804) study.

In both SURMOUNT-1 and SURMOUNT-2, tirzepatide was associated with reduced Cr-eGFR at week 24 compared with placebo. After week 24, no further reductions in eGFR were observed in SURMOUNT-2, whereas eGFR increased toward baseline in SURMOUNT-1. Similar eGFR trajectories have been observed in other studies with tirzepatide and semaglutide. We previously reported that the initial eGFR dip during tirzepatide treatment was partly reversible after discontinuation of tirzepatide in adults with type 2 diabetes at high cardiovascular risk, suggesting that the initial dip reflects a hemodynamic effect due to reduction in intraglomerular pressure.^3^ However, GFR and renal blood flow measurements using exogenous tracers are necessary to validate this notion.

Changes in Cys-C-eGFR followed a different pattern than Cr-eGFR, although comparisons are complicated since cystatin C was only measured twice during follow-up. Nevertheless, in contrast to Cr-eGFR, Cys-C-eGFR showed a marked increase during follow-up in participants in SURMOUNT-1. In SURMOUNT-2, there was no difference in Cys-C-eGFR between tirzepatide and placebo. Similar differences between participants with and without diabetes in Cys-C-eGFR trajectories have also been observed with retatrutide, a combined GLP-1, GIP, and glucagon RA.^33^ Prospective studies such as TREASURE-CKD that characterize the effects of tirzepatide on iohexol-measured GFR may aid in elucidating differences of tirzepatide on kidney function in people with obesity with or without diabetes.^34^

A clinically relevant question is whether creatinine and/or cystatin C can be used to estimate GFR in the setting of significant weight loss. Nonrenal determinants of creatinine and cystatin C are affected by muscle mass and fat mass, respectively.^35^ Tirzepatide reduced body weight up to 23% in the SURMOUNT-1 and up to 16% in the SURMOUNT-2 trials.^16,17^ Imaging studies demonstrated that participants treated with tirzepatide had a three times greater percent reduction in fat mass compared with lean mass.^17^ Our correlation analyses demonstrated no differences between changes in Cr-eGFR and body weight during tirzepatide treatment in SURMOUNT-1 and SURMOUNT-2. Similar findings have been reported in a tirzepatide study in participants with type 2 diabetes at high cardiovascular risk.^3^ Cystatin-C-eGFR changes correlated with body weight changes during treatment with tirzepatide in SURMOUNT-2. However, a similar correlation was observed during placebo treatment, making it difficult to assess whether this correlation is specifically attributed to tirzepatide-induced weight loss or due to other factors.

The strength of this study includes the large sample size, which allowed for precise estimation of the effect size of tirzepatide compared with placebo on UACR. This study also has limitations. First, most participants enrolled in the SURMOUNT-1 and SURMOUNT-2 trials did not have CKD; the proportion of participants with eGFR of <60 ml/min per 1.73 m^2^ was 2.0% and 6.2% in SURMOUNT-1 and SURMOUNT-2, respectively, which does not extrapolate the current findings to a population with impaired kidney function. As a result, only modest eGFR loss was observed in the placebo group during the 72 weeks of follow-up. Therefore, whether tirzepatide can attenuate eGFR decline requires additional studies with longer follow-up. Second, the lack of a gold standard–measured GFR assessment leaves the question as to how to optimally monitor GFR over time during treatment with tirzepatide. In addition, as this study was a *post hoc* exploratory analysis, chance findings cannot be excluded, and the results are therefore preliminary and can only be regarded as hypothesis-generating.

In conclusion, treatment with tirzepatide in people with obesity or overweight with or without type 2 diabetes was associated with preserved kidney function and reduced albuminuria. These associations were consistent in participants already using RAS inhibitors or SGLT2i. Tirzepatide was generally well tolerated and did not have a clear effect on eGFR. Whether tirzepatide would benefit kidney function in people with preexisting CKD requires a dedicated study.

## Supplementary Material

## Data Availability

This study includes clinical experimentation and received Institutional Review Board or Ethics Committee approval. All patients provided written informed consent. This study includes clinical experimentation and complies with the Declaration of Helsinki.

## Data Sharing Statement

Previously published data were used for this study; partial restrictions to the data and/or materials apply. Jastreboff AM, *et al.* *N Engl J Med*. 2022; 387:205–216. Garvey WT, *et al.* *Lancet* 2023; 402:613–626. Lilly provides access to all individual participant data collected during the trial, after anonymization, with the exception of pharmacokinetic or genetic data. Data are available to request 6 months after the indication studied has been approved in the United States and European Union and after primary publication acceptance, whichever is later. No expiration date of data requests is currently set once data are made available. Access is provided after a proposal has been approved by an independent review committee identified for this purpose and after receipt of a signed data sharing agreement. Data and documents, including the study protocol, statistical analysis plan, clinical study report, blank or annotated case report forms, will be provided in a secure data sharing environment. For details on submitting a request, see the instructions provided at www.vivli.org.

## Supplemental Material

Supplemental Figure 1. SURMOUNT-1 subgroup analyses of the association of tirzepatide compared with placebo with eGFR changes with baseline eGFR <60 or ≥60 ml/min per 1.73 m^2^ or baseline UACR <30 or ≥30 mg/g, and association of tirzepatide compared with placebo with UACR changes with various baseline characteristics.

Supplemental Figure 2. SURMOUNT-2 subgroup analyses of the association of tirzepatide compared with placebo with eGFR changes with baseline eGFR <60 or ≥60 ml/min per 1.73 m^2^ or baseline UACR <30 or ≥30 mg/g, and association of tirzepatide compared with placebo with UACR changes with various baseline characteristics.

Supplemental Figure 3. Correlation between change in body weight and change in eGFR for SURMOUNT-1.

Supplemental Figure 4. Correlation between change in body weight and change in eGFR for SURMOUNT-2.

Supplemental Table 1. Summary of SURMOUNT-1 and SURMOUNT-2 studies.

Supplemental Table 2. Safety overview.

Supplemental Table 3. Percent change from baseline at week 72 in UACR by tirzepatide dose for SURMOUNT-1 and SURMOUNT-2.

Supplemental Table 4. Change from baseline at week 72 in nonindexed eGFR for SURMOUNT-1 and SURMOUNT-2.
