# Supplementary material for: Kidney Parameters with Tirzepatide in Obesity with or without Type 2 Diabetes
Source: J Am Soc Nephrol. 2025 Jun 13;36(11):2190–200. doi: 10.1681/ASN.0000000764 (PMC12591676; doi:10.1681/ASN.0000000764)
Supplement: Supplementary file 1 [file jasn-36-2190-s001.pdf]

ASN American Society of Nephrology
JASN Journal of the American Society of Nephrology
KIDNEY360 Accessing Our World From Every Angle
CJASN Clinical Journal of the American Society of Nephrology

ASN Journal Disclosure Form

As per ASN journal policy, I have disclosed any financial relationships or commitments I have held in the past 36 months as included below. I have listed my Current Employer below to indicate there is a relationship requiring disclosure. If no relationship exists, my Current Employer is not listed.

I. Benabbad reports the following:
Employer: Eli Lilly; and Ownership Interest: Eli Lilly.

I understand that the information above will be published within the journal article, if accepted, and that failure to comply and/or to accurately and completely report the potential financial conflicts of interest could lead to the following: 1) Prior to publication, article rejection, or 2) Post-publication, sanctions ranging from, but not limited to, issuing a correction, reporting the inaccurate information to the authors' institution, banning authors from submitting work to ASN journals for varying lengths of time, and/or retraction of the published work.

Name: Imane Benabbad
Manuscript ID: JASN-2025-000023R1
Manuscript Title: Kidney Parameters with Tirzepatide in Obesity with or without Type 2 Diabetes: Results from SURMOUNT-1 and -2
Date of Completion: May 26, 2025
Disclosure Updated Date: May 26, 2025

ASN American Society of Nephrology | JASN Journal of the American Society of Nephrology | KIDNEY360 Accessing Our World From Every Angle | CJASN Clinical Journal of the American Society of Nephrology

ASN Journal Disclosure Form

As per ASN journal policy, I have disclosed any financial relationships or commitments I have held in the past 36 months as included below. I have listed my Current Employer below to indicate there is a relationship requiring disclosure. If no relationship exists, my Current Employer is not listed.

P. Bjornstad reports the following:
Employer: University of Washington; Consultancy: AstraZeneca; Boehringer Ingelheim; Bayer; Horizon Pharma; XORTX; Novo Nordisk; Lilly; Research Funding: Horizon Pharma/Amgen, AstraZeneca, Merck, Novo Nordisk, Lilly; and Advisory or Leadership Role: Horizon Pharma/Amgen, XORTX, AstraZeneca, Boehringer-Ingelheim, Bayer, Lilly, Novo Nordisk.

I understand that the information above will be published within the journal article, if accepted, and that failure to comply and/or to accurately and completely report the potential financial conflicts of interest could lead to the following: 1) Prior to publication, article rejection, or 2) Post-publication, sanctions ranging from, but not limited to, issuing a correction, reporting the inaccurate information to the authors' institution, banning authors from submitting work to ASN journals for varying lengths of time, and/or retraction of the published work.

Name: Petter Bjornstad
Manuscript ID: JASN-2025-000023R1
Manuscript Title: Kidney Parameters with Tirzepatide in Obesity with or without Type 2 Diabetes: Results from SURMOUNT-1 and -2
Date of Completion: May 24, 2025
Disclosure Updated Date: February 17, 2025

ASN American Society of Nephrology | JASN Journal of the American Society of Nephrology | KIDNEY360 Accessing Our World From Every Angle | CJASN Clinical Journal of the American Society of Nephrology

ASN Journal Disclosure Form

As per ASN journal policy, I have disclosed any financial relationships or commitments I have held in the past 36 months as included below. I have listed my Current Employer below to indicate there is a relationship requiring disclosure. If no relationship exists, my Current Employer is not listed.

M. Bunck reports the following:
Employer: Eli Lilly and Company; and Ownership Interest: Eli Lilly and Company.

I understand that the information above will be published within the journal article, if accepted, and that failure to comply and/or to accurately and completely report the potential financial conflicts of interest could lead to the following: 1) Prior to publication, article rejection, or 2) Post-publication, sanctions ranging from, but not limited to, issuing a correction, reporting the inaccurate information to the authors' institution, banning authors from submitting work to ASN journals for varying lengths of time, and/or retraction of the published work.

Name: Mathijs Bunck
Manuscript ID: JASN-2025-000023R1
Manuscript Title: Kidney Parameters with Tirzepatide in Obesity with or without Type 2 Diabetes: Results from SURMOUNT-1 and -2
Date of Completion: May 23, 2025
Disclosure Updated Date: May 6, 2025

ASN American Society of Nephrology
JASN Journal of the American Society of Nephrology
KIDNEY360 Accessing Our World From Every Angle
CJASN Clinical Journal of the American Society of Nephrology

ASN Journal Disclosure Form

As per ASN journal policy, I have disclosed any financial relationships or commitments I have held in the past 36 months as included below. I have listed my Current Employer below to indicate there is a relationship requiring disclosure. If no relationship exists, my Current Employer is not listed.

D. Cao reports the following:
Employer: Eli Lilly and Company; and Other Interests or Relationships: Stock holder of Eli Lilly and Company.

I understand that the information above will be published within the journal article, if accepted, and that failure to comply and/or to accurately and completely report the potential financial conflicts of interest could lead to the following: 1) Prior to publication, article rejection, or 2) Post-publication, sanctions ranging from, but not limited to, issuing a correction, reporting the inaccurate information to the authors' institution, banning authors from submitting work to ASN journals for varying lengths of time, and/or retraction of the published work.

Name: Dachuang Cao
Manuscript ID: JASN-2025-000023R1
Manuscript Title: Kidney Parameters with Tirzepatide in Obesity with or without Type 2 Diabetes: Results from SURMOUNT-1 and -2
Date of Completion: May 23, 2025
Disclosure Updated Date: May 23, 2025

ASN American Society of Nephrology
JASN Journal of the American Society of Nephrology
KIDNEY360 Accessing Our World From Every Angle
CJASN Clinical Journal of the American Society of Nephrology

ASN Journal Disclosure Form

As per ASN journal policy, I have disclosed any financial relationships or commitments I have held in the past 36 months as included below. I have listed my Current Employer below to indicate there is a relationship requiring disclosure. If no relationship exists, my Current Employer is not listed.

D. Cherney reports the following:
Employer: Toronto General Hospital; Consultancy: Boehringer Ingelheim-Lilly, Merck, AstraZeneca, Sanofi, Mitsubishi-Tanabe, Abbvie, Janssen, AMGEN, Bayer, Prometic, BMS, Maze, Gilead, CSL-Behring, Otsuka, Novartis, Youngene, Lexicon, Inversago, GSK, Biobridge, Vantage, Altimmune and Novo-Nordisk; Research Funding: Boehringer Ingelheim-Lilly, Merck, Janssen, Sanofi, AstraZeneca, CSL-Behring, Lexicon, Novo-Nordisk, Bayer.; Honoraria: Boehringer Ingelheim-Lilly, Merck, AstraZeneca, Sanofi, Mitsubishi-Tanabe, Abbvie, Janssen, AMGEN, Bayer, Prometic, BMS, Maze, Gilead, CSL-Behring, Otsuka, Novartis, Youngene, Lexicon, Inversago, GSK, Biobridge, Vantage, Altimmune and Novo-Nordisk; and Advisory or Leadership Role: Boehringer Ingelheim-Lilly, Merck, AstraZeneca, Lexicon, Janssen, Bayer, BMS, Maze, CSL-Behring, Novartis, Novo-Nordisk.

I understand that the information above will be published within the journal article, if accepted, and that failure to comply and/or to accurately and completely report the potential financial conflicts of interest could lead to the following: 1) Prior to publication, article rejection, or 2) Post-publication, sanctions ranging from, but not limited to, issuing a correction, reporting the inaccurate information to the authors' institution, banning authors from submitting work to ASN journals for varying lengths of time, and/or retraction of the published work.

Name: David Cherney
Manuscript ID: JASN-2025-000023R1
Manuscript Title: Kidney Parameters with Tirzepatide in Obesity with or without Type 2 Diabetes: Results from SURMOUNT-1 and -2
Date of Completion: May 22, 2025
Disclosure Updated Date: January 7, 2025

ASN
American Society of Nephrology
JASN
Journal of the American Society of Nephrology
KIDNEY360
Accessing Our World From Every Angle
CJASN
Clinical Journal of the American Society of Nephrology

ASN Journal Disclosure Form

As per ASN journal policy, I have disclosed any financial relationships or commitments I have held in the past 36 months as included below. I have listed my Current Employer below to indicate there is a relationship requiring disclosure. If no relationship exists, my Current Employer is not listed.

A. Friedman reports the following:
Employer: Indiana University School of Medicine; Consultancy: Gila Therapeutics; Morphic Medical; Eli Lilly; Novo Nordisk; Ownership Interest: Gila Therapeutics; and Advisory or Leadership Role: Scientific Advisory Board: Morphic Medical (paid), Gila Therapeutics(paid), Eli Lilly(paid); Novo Nordisk (paid); Editorial Board: Journal of Renal Nutrition, Frontiers in Nephrology; ; Council member: International Society of Renal Nutrition and Metabolism.

I understand that the information above will be published within the journal article, if accepted, and that failure to comply and/or to accurately and completely report the potential financial conflicts of interest could lead to the following: 1) Prior to publication, article rejection, or 2) Post-publication, sanctions ranging from, but not limited to, issuing a correction, reporting the inaccurate information to the authors' institution, banning authors from submitting work to ASN journals for varying lengths of time, and/or retraction of the published work.

Name: Allon N. Friedman
Manuscript ID: JASN-2025-000023R2
Manuscript Title: Kidney Parameters with Tirzepatide in Obesity with or without Type 2 Diabetes
Date of Completion: May 23, 2025
Disclosure Updated Date: May 23, 2025

ASN American Society of Nephrology | JASN Journal of the American Society of Nephrology | KIDNEY360 Accessing Our World From Every Angle | CJASN Clinical Journal of the American Society of Nephrology

ASN Journal Disclosure Form

As per ASN journal policy, I have disclosed any financial relationships or commitments I have held in the past 36 months as included below. I have listed my Current Employer below to indicate there is a relationship requiring disclosure. If no relationship exists, my Current Employer is not listed.

L. Garcia reports the following:
Employer: Eli Lilly and Company; Ownership Interest: Eli Lilly and Company; and Honoraria: Eli Lilly and Company.

I understand that the information above will be published within the journal article, if accepted, and that failure to comply and/or to accurately and completely report the potential financial conflicts of interest could lead to the following: 1) Prior to publication, article rejection, or 2) Post-publication, sanctions ranging from, but not limited to, issuing a correction, reporting the inaccurate information to the authors' institution, banning authors from submitting work to ASN journals for varying lengths of time, and/or retraction of the published work.

Name: Luis Emilio Garcia
Manuscript ID: JASN-2025-000023R1
Manuscript Title: Kidney Parameters with Tirzepatide in Obesity with or without Type 2 Diabetes: Results from SURMOUNT-1 and -2
Date of Completion: May 26, 2025
Disclosure Updated Date: May 26, 2025

ASN American Society of Nephrology
JASN Journal of the American Society of Nephrology
KIDNEY360 Accessing Our World From Every Angle
CJASN Clinical Journal of the American Society of Nephrology

ASN Journal Disclosure Form

As per ASN journal policy, I have disclosed any financial relationships or commitments I have held in the past 36 months as included below. I have listed my Current Employer below to indicate there is a relationship requiring disclosure. If no relationship exists, my Current Employer is not listed.

R. Griffin reports the following:
Employer: Eli Lilly; and Ownership Interest: Eli Lilly.

I understand that the information above will be published within the journal article, if accepted, and that failure to comply and/or to accurately and completely report the potential financial conflicts of interest could lead to the following: 1) Prior to publication, article rejection, or 2) Post-publication, sanctions ranging from, but not limited to, issuing a correction, reporting the inaccurate information to the authors' institution, banning authors from submitting work to ASN journals for varying lengths of time, and/or retraction of the published work.

Name: Ryan Griffin
Manuscript ID: JASN-2025-000023R1
Manuscript Title: Kidney Parameters with Tirzepatide in Obesity with or without Type 2 Diabetes: Results from SURMOUNT-1 and -2
Date of Completion: May 22, 2025
Disclosure Updated Date: May 22, 2025

ASN American Society of Nephrology · JASN Journal of the American Society of Nephrology · KIDNEY360 Accessing Our World From Every Angle · CJASN Clinical Journal of the American Society of Nephrology

ASN Journal Disclosure Form

As per ASN journal policy, I have disclosed any financial relationships or commitments I have held in the past 36 months as included below. I have listed my Current Employer below to indicate there is a relationship requiring disclosure. If no relationship exists, my Current Employer is not listed.

H. Heerspink reports the following:
Employer: University Medical Center Groningen; Consultancy: Ongoing consultancy agreements with AstraZeneca, Alexion, Bayer, Boehringer Ingelheim, Biocity Therapeutic, CSL Behring, Dimerix, Eli-Lilly, Gilead, Idorsia, Janssen, Novartis, NovoNordisk, Roche, Travere Therapeutics; Research Funding: AstraZeneca, Bayer, Boehringer Ingelheim, NovoNordisk and Janssen research support (grant funding directed to employer); Honoraria: Lecture fees from AstraZeneca and NovoNordisk; and Speakers Bureau: AstraZeneca.

I understand that the information above will be published within the journal article, if accepted, and that failure to comply and/or to accurately and completely report the potential financial conflicts of interest could lead to the following: 1) Prior to publication, article rejection, or 2) Post-publication, sanctions ranging from, but not limited to, issuing a correction, reporting the inaccurate information to the authors' institution, banning authors from submitting work to ASN journals for varying lengths of time, and/or retraction of the published work.

Name: Hiddo Jan L. Heerspink
Manuscript ID: JASN-2025-000023R1
Manuscript Title: Kidney Parameters with Tirzepatide in Obesity with or without Type 2 Diabetes: Results from SURMOUNT-1 and -2
Date of Completion: May 5, 2025
Disclosure Updated Date: January 2, 2025

ASN American Society of Nephrology
JASN Journal of the American Society of Nephrology
KIDNEY360 Accessing Our World From Every Angle
CJASN Clinical Journal of the American Society of Nephrology

ASN Journal Disclosure Form

As per ASN journal policy, I have disclosed any financial relationships or commitments I have held in the past 36 months as included below. I have listed my Current Employer below to indicate there is a relationship requiring disclosure. If no relationship exists, my Current Employer is not listed.

C. Piras de Oliveira reports the following:
Employer: Neurocrine Biosciences; Eli Lilly and Company; Ownership Interest: Eli Lilly and Company; Neurocrine Biosciences; and Honoraria: Eli Lilly and Company; Neurocrine Biosciences.

I understand that the information above will be published within the journal article, if accepted, and that failure to comply and/or to accurately and completely report the potential financial conflicts of interest could lead to the following: 1) Prior to publication, article rejection, or 2) Post-publication, sanctions ranging from, but not limited to, issuing a correction, reporting the inaccurate information to the authors' institution, banning authors from submitting work to ASN journals for varying lengths of time, and/or retraction of the published work.

Name: Carolina Piras de Oliveira
Manuscript ID: JASN-2025-000023R1
Manuscript Title: Kidney Parameters with Tirzepatide in Obesity with or without Type 2 Diabetes: Results from SURMOUNT-1 and -2
Date of Completion: May 23, 2025
Disclosure Updated Date: May 23, 2025

ASN American Society of Nephrology
JASN Journal of the American Society of Nephrology
KIDNEY360 Accessing Our World From Every Angle
CJASN Clinical Journal of the American Society of Nephrology

ASN Journal Disclosure Form

As per ASN journal policy, I have disclosed any financial relationships or commitments I have held in the past 36 months as included below. I have listed my Current Employer below to indicate there is a relationship requiring disclosure. If no relationship exists, my Current Employer is not listed.

A. Stefanski reports the following:
Employer: Eli Lilly; and Ownership Interest: Eli Lilly.

I understand that the information above will be published within the journal article, if accepted, and that failure to comply and/or to accurately and completely report the potential financial conflicts of interest could lead to the following: 1) Prior to publication, article rejection, or 2) Post-publication, sanctions ranging from, but not limited to, issuing a correction, reporting the inaccurate information to the authors' institution, banning authors from submitting work to ASN journals for varying lengths of time, and/or retraction of the published work.

Name: Adam Stefanski
Manuscript ID: JASN-2025-000023R2
Manuscript Title: Kidney Parameters with Tirzepatide in Obesity with or without Type 2 Diabetes
Date of Completion: May 23, 2025
Disclosure Updated Date: May 23, 2025

ASN American Society of Nephrology
JASN Journal of the American Society of Nephrology
KIDNEY360 Accessing Our World From Every Angle
CJASN Clinical Journal of the American Society of Nephrology

ASN Journal Disclosure Form

As per ASN journal policy, I have disclosed any financial relationships or commitments I have held in the past 36 months as included below. I have listed my Current Employer below to indicate there is a relationship requiring disclosure. If no relationship exists, my Current Employer is not listed.

I. Turfanda reports the following:
Employer: Eli Lilly and Company; and Ownership Interest: Eli Lilly and Company.

I understand that the information above will be published within the journal article, if accepted, and that failure to comply and/or to accurately and completely report the potential financial conflicts of interest could lead to the following: 1) Prior to publication, article rejection, or 2) Post-publication, sanctions ranging from, but not limited to, issuing a correction, reporting the inaccurate information to the authors' institution, banning authors from submitting work to ASN journals for varying lengths of time, and/or retraction of the published work.

Name: Ibrahim Turfanda
Manuscript ID: JASN-2025-000023R1
Manuscript Title: Kidney Parameters with Tirzepatide in Obesity with or without Type 2 Diabetes: Results from SURMOUNT-1 and -2,
Date of Completion: May 5, 2025
Disclosure Updated Date: May 5, 2025

ASN
American Society of Nephrology
JASN
Journal of the American Society of Nephrology
KIDNEY360
Accessing Our World From Every Angle
CJASN
Clinical Journal of the American Society of Nephrology

ASN Journal Disclosure Form

As per ASN journal policy, I have disclosed any financial relationships or commitments I have held in the past 36 months as included below. I have listed my Current Employer below to indicate there is a relationship requiring disclosure. If no relationship exists, my Current Employer is not listed.

D. van Raalte reports the following:
Employer: Amsterdam University Medical Center; Consultancy: Astra Zeneca; Bayer; Boehringer Ingelheim, Ely Lilly; Merck, Novo Nordisk; and Research Funding: Astra Zeneca; Boehringer Ingelheim , Ely Lilly; Merck, Novo Nordisk.

I understand that the information above will be published within the journal article, if accepted, and that failure to comply and/or to accurately and completely report the potential financial conflicts of interest could lead to the following: 1) Prior to publication, article rejection, or 2) Post-publication, sanctions ranging from, but not limited to, issuing a correction, reporting the inaccurate information to the authors' institution, banning authors from submitting work to ASN journals for varying lengths of time, and/or retraction of the published work.

Name: Daniël H. van Raalte
Manuscript ID: JASN-2025-000023R1
Manuscript Title: Kidney Parameters with Tirzepatide in Obesity with or without Type 2 Diabetes: Results from SURMOUNT-1 and -2
Date of Completion: May 23, 2025
Disclosure Updated Date: May 23, 2025
