# Supplementary material for: Kidney Parameters with Tirzepatide in Obesity with or without Type 2 Diabetes
Source: J Am Soc Nephrol. 2025 Jun 13;36(11):2190–200. doi: 10.1681/ASN.0000000764 (PMC12591676; doi:10.1681/ASN.0000000764)
Supplement: Supplementary file 2 [file jasn-36-2190-s002.pdf]

**SUPPLEMENTARY MATERIAL**

# Kidney Parameters with Tirzepatide in Obesity with or without Type 2 Diabetes

## Kidney Parameters with Tirzepatide in SURMOUNT Trials

*Hiddo J. L. Heerspink[1], PhD; Allon N. Friedman[2], MD; Petter Bjornstad[3], MD; Daniel H. van Raalte[4], MD, PhD; David Cherney[5], MD, PhD; Dachuang Cao[6], PhD; Luis-Emilio Garcia-Pérez[6], MD, PhD; Adam Stefanski[6], MD, PhD; Ibrahim Turfanda[6], MD, MSc; Mathijs C. Bunck[6], MD PhD; Imane Benabbad[6], MD; Ryan Griffin[6], PharmD; Carolina Piras de Oliveira*[,6], MD*

[1]Department of Clinical Pharmacy, University of Groningen, University Medical Center Groningen, Groningen, The Netherlands

[2]Indiana University School of Medicine, Indianapolis, Indiana, USA

[3]University of Washington School of Medicine, Seattle, Washington, USA

[4]Amsterdam University Medical Center, Amsterdam, The Netherlands

[5]Department of Medicine, Toronto General Hospital Research Institute, University Health Network, Toronto, Ontario, Canada

[6]Eli Lilly and Company, Indianapolis, Indiana, USA

*Affiliated with Eli Lilly and Company, Indianapolis, Indiana, USA at the time of the study

**Corresponding author information**

Hiddo J. L. Heerspink, PhD

University of Groningen,

Hanzeplein 1, PO Box 30 001,

Groningen, the Netherlands

Tel: +31 5036 14071

email: h.j.lambers.heerspink@umcg.nl

**Table of Contents**

Supplemental Figure 1. SURMOUNT-1 subgroup analyses of the association of tirzepatide compared with placebo with eGFR changes with baseline eGFR <60 or ≥60 ml/min per 1.73 $m^2$ or baseline UACR <30 mg/g or ≥30 mg/g, and association of tirzepatide compared with placebo with UACR changes with various baseline characteristics.................................3

Supplemental Figure 2. SURMOUNT-2 subgroup analyses of the association of tirzepatide compared with placebo with eGFR changes with baseline eGFR <60 or ≥60 ml/min per 1.73 $m^2$ or baseline UACR <30 mg/g or ≥30 mg/g, and association of tirzepatide compared with placebo with UACR changes with various baseline characteristics.................................4

Supplemental Figure 3. Correlation between change in body weight and change in eGFR for SURMOUNT-1.........................................................................................................................5

Supplemental Figure 4. Correlation between change in body weight and change in eGFR for SURMOUNT-2.........................................................................................................................6

Supplemental Table 1. Summary of SURMOUNT-1 and SURMOUNT-2 studies....................7

Supplemental Table 2. Safety overview.......................................................................................8

Supplemental Table 3. Percent change from baseline at week 72 in UACR by tirzepatide dose for SURMOUNT-1 and SURMOUNT-2..............................................................................9

Supplemental Table 4. Change from baseline at week 72 in non-indexed eGFR for SURMOUNT-1 and SURMOUNT-2.........................................................................................10

**Supplemental Figure 1. SURMOUNT-1 subgroup analyses of the association of tirzepatide compared with placebo with eGFR changes with baseline eGFR <60 or ≥60 ml/min per 1.73 m$^2$ or baseline UACR <30 mg/g or ≥30 mg/g, and association of tirzepatide compared with placebo with UACR changes with various baseline characteristics**. ACEi, angiotensin-converting enzyme inhibitor; ARB, angiotensin receptor blocker; BMI, body mass index; CI, confidence interval; Cr-Cys-C-eGFR, creatinine-cystatin-C-based estimated glomerular filtration rate; Cys-C-eGFR, cystatin-C-based estimated glomerular filtration rate; eGFR, estimated glomerular filtration rate; ETD, estimated treatment difference; LSM, least squares mean; SBP, systolic blood pressure; UACR, urine albumin-to-creatinine ratio.

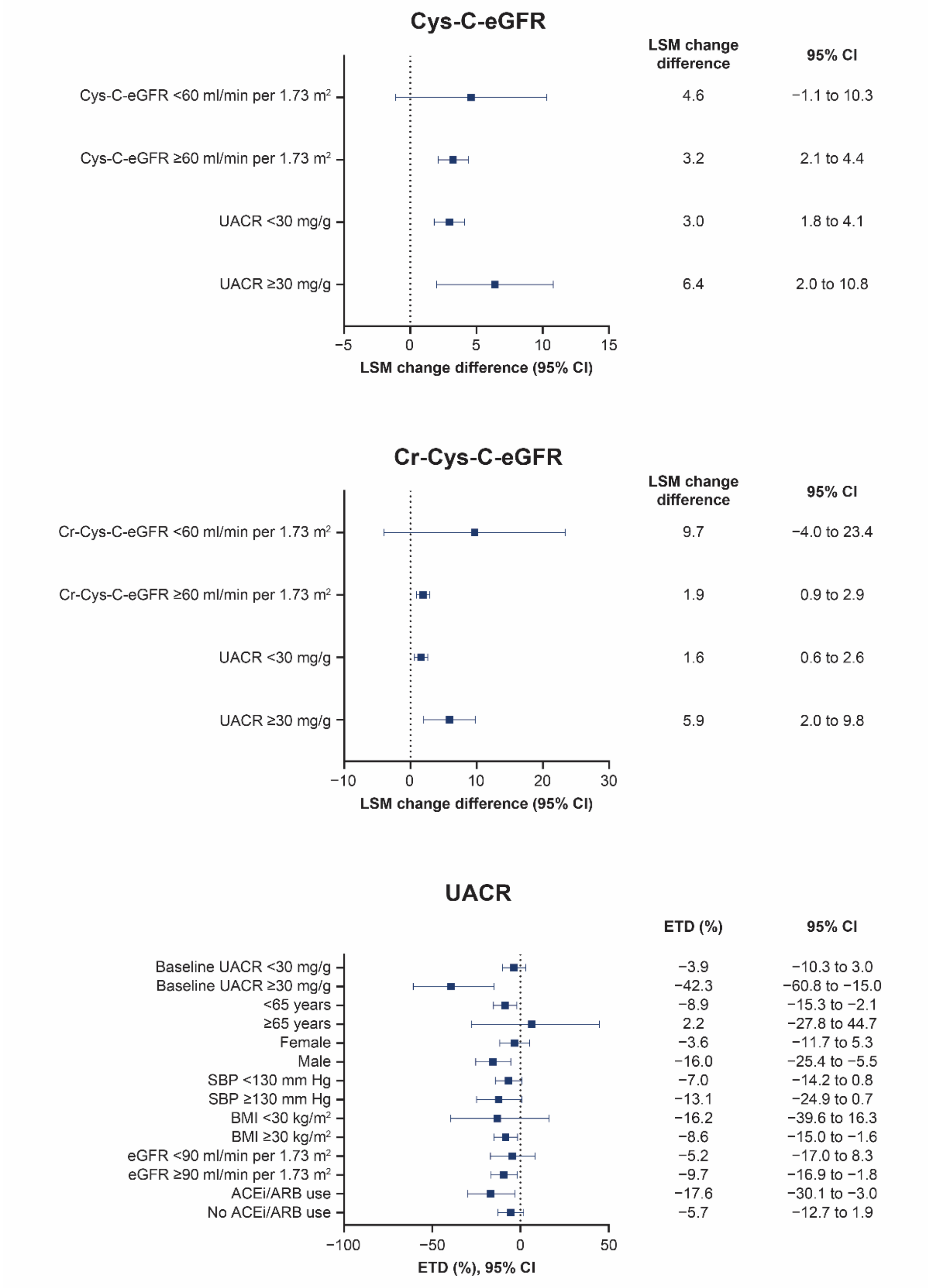

**Supplemental Figure 2. SURMOUNT-2 subgroup analyses of the association of tirzepatide compared with placebo with eGFR changes with baseline eGFR <60 or ≥60 ml/min per 1.73 m$^2$ or baseline UACR <30 mg/g or ≥30 mg/g, and association of tirzepatide compared with placebo with UACR changes with various baseline characteristics.** ACEi, angiotensin-converting enzyme inhibitor; ARB, angiotensin receptor blocker; BMI, body mass index; CI, confidence interval; Cr-Cys-C-eGFR, creatinine-cystatin-C-based estimated glomerular filtration rate; Cys-C-eGFR, cystatin-C-based estimated glomerular filtration rate; ETD, estimated treatment difference; LSM, least squares mean; eGFR, estimated glomerular filtration rate; SBP, systolic blood pressure; SGLT-2i, sodium-glucose co-transporter-2 inhibitor; UACR, urine albumin-to-creatinine ratio.

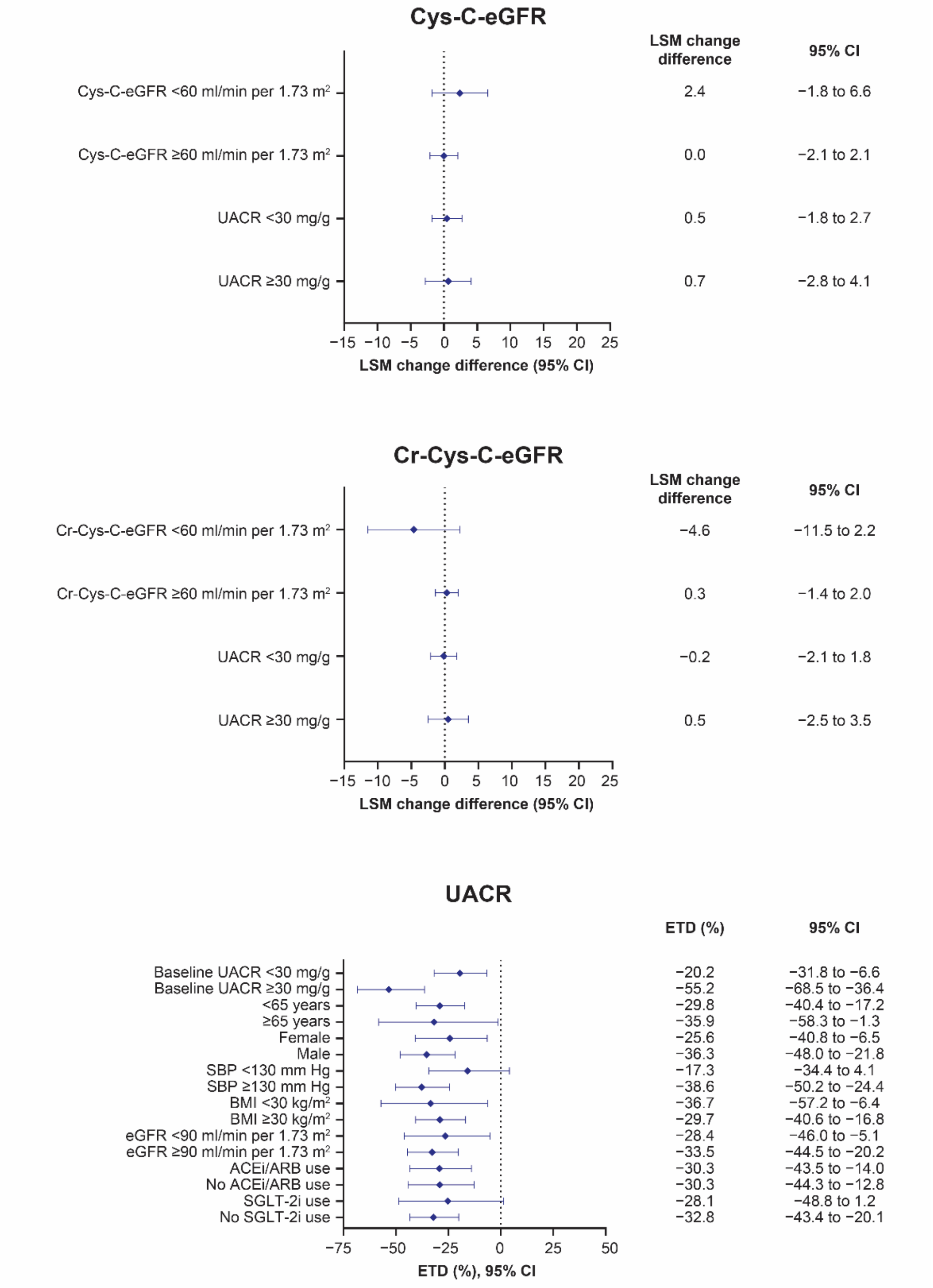

**Supplemental Figure 3. Correlation between change in body weight and change in eGFR for SURMOUNT-1.** Note: Relationships between change in body weight and change in eGFR were analyzed using Pearson correlation. Cr-Cys-C-eGFR, creatinine-cystatin-C-based estimated glomerular filtration rate; Cr-eGFR, creatinine-based estimated glomerular filtration rate; Cys-C-eGFR, cystatin-C-based estimated glomerular filtration rate; eGFR, estimated glomerular filtration rate; TZP, tirzepatide.

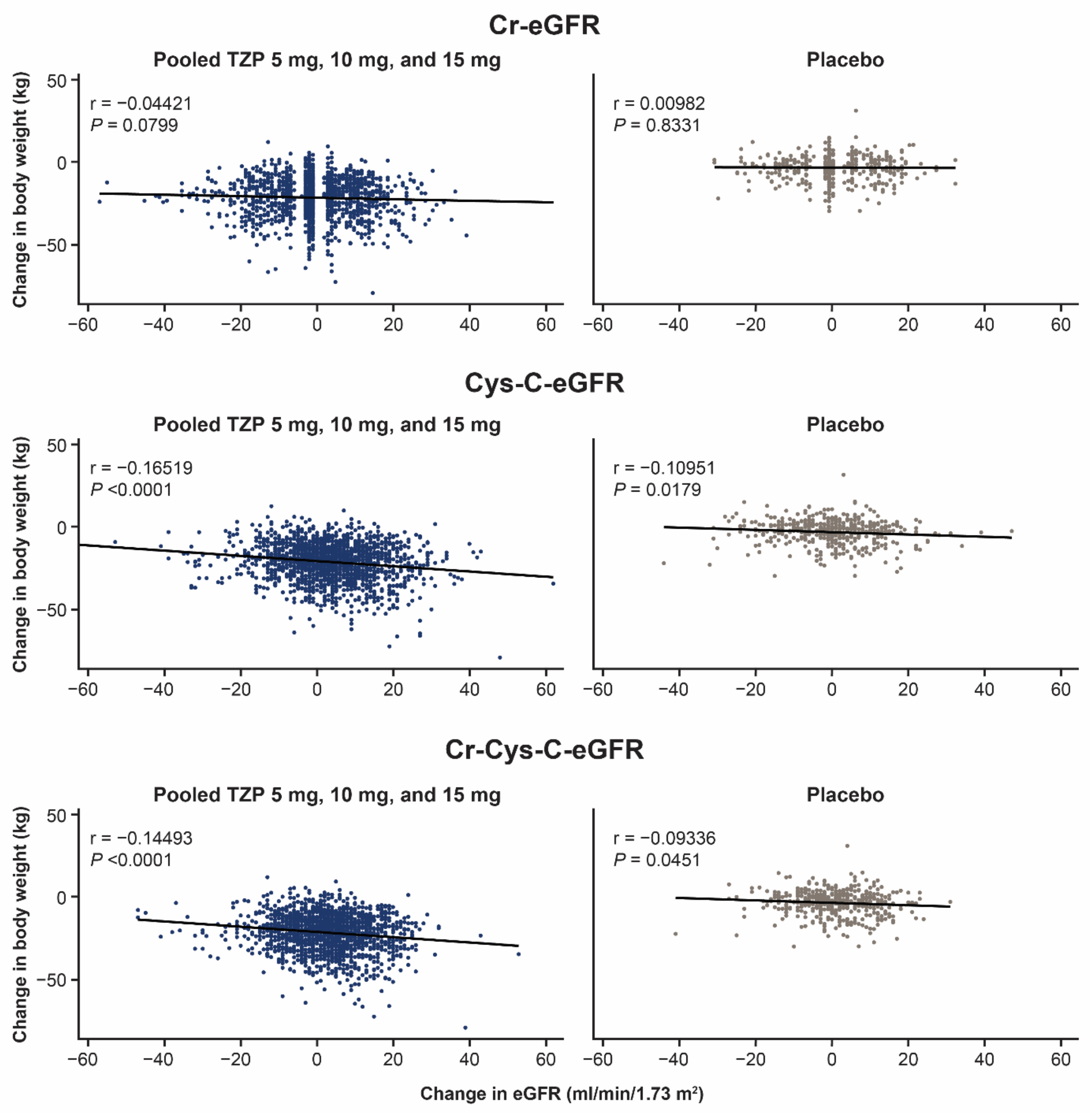

**Supplemental Figure 4. Correlation between change in body weight and change in eGFR for SURMOUNT-2.** Note: Relationships between change in body weight and change in eGFR were analyzed using Pearson correlation. Cr-Cys-C-eGFR, creatinine-cystatin-C-based estimated glomerular filtration rate; Cr-eGFR, creatinine-based estimated glomerular filtration rate; Cys-C-eGFR, cystatin-C-based estimated glomerular filtration rate; eGFR, estimated glomerular filtration rate; TZP, tirzepatide.

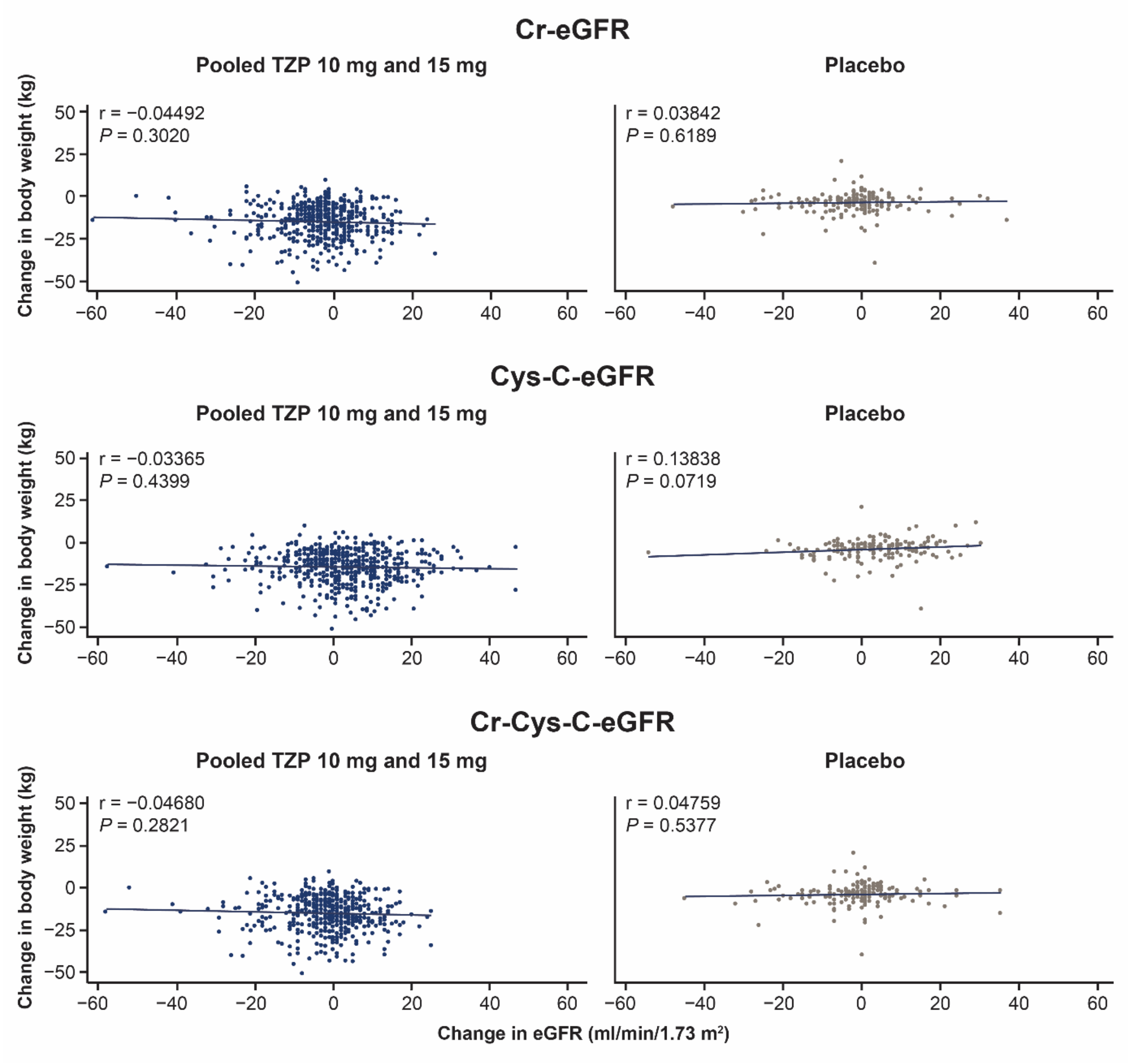

**Supplemental Table 1. Summary of SURMOUNT-1 and SURMOUNT-2 studies**

| Trial characteristic | SURMOUNT-1 | SURMOUNT-2 |
|---|---|---|
| Trial design | Phase 3, double-blind, placebo-controlled RCT | Phase 3, double-blind, placebo-controlled RCT |
| Trial duration | 72 weeks | 72 weeks |
| Coprimary outcome | Percent change in body weight from baseline to week 72 and percentage of participants with a weight reduction of ≥5% from baseline at week 72 | Percent change in body weight from baseline to week 72 and percentage of participants with a weight reduction of ≥5% from baseline at week 72 |
| Treatment arms | TZP 5 mg QW<br>TZP 10 mg QW<br>TZP 15 mg QW<br>PBO | TZP 10 mg QW<br>TZP 15 mg QW<br>PBO |
| Key inclusion criteria | • ≥18 years<br>• BMI ≥30 kg/$m^2$ or ≥27 kg/$m^2$ with ≥1 weight-related complication (eg, hypertension, dyslipidemia, obstructive sleep apnea, or cardiovascular disease)<br>• Reported ≥1 unsuccessful dietary effort to lose weight | • ≥18 years<br>• BMI ≥27 kg/$m^2$<br>• T2D with HbA1c of 7–10% (53–86 mmol/mol)<br>• On stable therapy for T2D for ≥3 months with diet or exercise alone or any oral agent (except DPP-4) inhibitors or GLP-1 receptor agonists |
| Key exclusion criteria | • T1D or T2D<br>• eGFR <30 ml/min per 1.73 $m^2$<br>• Change in body weight of ≥5 kg within 3 months before screening<br>• Previous or planned surgical treatment for obesity<br>• Treatment with a medication that promotes weight loss within 3 months before screening | • eGFR <30 ml/min per 1.73 $m^2$<br>• Change in body weight of ≥5 kg within 3 months before screening<br>• Previous or planned surgical treatment for obesity<br>• Treatment with anti-obesity medications, DPP-4 inhibitors, oral GLP-1 receptor agonists, or any injectable therapy for T2D within 3 months of screening |

BMI, body mass index; DPP-4, dipeptidyl peptidate-4; eGFR, estimated glomerular filtration rate; GLP-1, glucagon-like peptide-1; HbA1c, glycated hemoglobin; PBO, placebo; QW, once weekly; RCT, randomized controlled trial; T1D, type 1 diabetes; T2D, type 2 diabetes; TZP, tirzepatide.

**Supplemental Table 2. Safety overview**

| TEAE, *n* (%) | SURMOUNT-1 | | | | SURMOUNT-2 | | |
|---|---|---|---|---|---|---|---|
| | PBO (*N*=643) | TZP 5 mg (*N*=630) | TZP 10 mg (*N*=636) | TZP 15 mg (*N*=630) | PBO (*N*=315) | TZP 10 mg (*N*=312) | TZP 15 mg (*N*=311) |
| Renal and urinary disorders | 16 (2.5) | 17 (2.7) | 15 (2.4) | 15 (2.4) | 12 (3.8) | 16 (5.1) | 10 (3.2) |
| Acute kidney injury | 1 (0.2) | 3 (0.5) | 2 (0.3) | 4 (0.6) | 1 (0.3) | 4 (1.3) | 0 |
| Electrolytes | | | | | | | |
| Blood potassium increased | 0 | 2 (0.3) | 0 | 0 | 0 | 1 (0.3) | 0 |
| Blood potassium decreased | 1 (0.2) | 0 | 2 (0.3) | 1 (0.2) | – | – | – |
| Blood calcium decreased | – | – | – | – | 0 | 1 (0.3) | 0 |
| Blood alkaline phosphatase increased | 1 (0.2) | 3 (0.5) | 0 | 2 (0.3) | 1 (0.3) | 0 | 0 |
| Participants with ≥1 fractures | 8 (1.2) | 6 (1.0) | 7 (1.1) | 6 (1.0) | 4 (1.3) | 2 (0.6) | 6 (1.9) |
| Fractures and dislocations NEC | 1 (0.2) | 1 (0.2) | 3 (0.5) | 2 (0.3) | 1 (0.3) | 0 | 1 (0.3) |
| Limb fractures and dislocations | 6 (0.9) | 3 (0.5) | 3 (0.5) | 4 (0.6) | 2 (0.6) | 1 (0.3) | 5 (1.6) |
| Skull fractures, facial bone fractures, and dislocations | 1 (0.2) | 1 (0.2) | 0 | 0 | – | – | – |
| Spinal fractures and dislocations | 0 | 1 (0.2) | 1 (0.2) | 0 | – | – | – |
| Thoracic cage fractures and dislocations | 0 | 1 (0.2) | 0 | 0 | 1 (0.3) | 1 (0.3) | 0 |

NEC, not elsewhere classified; PBO, placebo; TEAE, treatment-emergent adverse event; TZP, tirzepatide.

**Supplemental Table 3. Percent change from baseline at week 72 in UACR by tirzepatide dose for SURMOUNT-1 and SURMOUNT-2**

| | SURMOUNT-1 | | | | SURMOUNT-2 | | |
|---|---|---|---|---|---|---|---|
| Treatment | PBO (*N*=643) | TZP 5 mg (*N*=630) | TZP 10 mg (*N*=636) | TZP 15 mg (*N*=630) | PBO (*N*=315) | TZP 10mg (*N*=312) | TZP 15 mg (*N*=311) |
| UACR | -3.6 (3.08) | -11.7 (2.66) | -14.4 (2.61) | -8.8 (2.76) | -20.9 (5.35) | -46.7 (2.95) | -44.2 (3.17) |
| ETD (95% CI) vs. PBO | | -8.4 (-16.0 to -0.2)* | -11.2 (-18.6 to -3.2)** | -5.4 (-13.2 to 3.1) | | -32.6 (-43.2 to -20.0)*** | -29.5 (-40.7 to -16.2)*** |
| UACR <30 mg/g | 3.0 (3.18) | -1.6 (2.81) | -3.0 (2.85) | 1.7 (2.96) | -9.1 (6.30) | -31.8 (3.78) | -22.6 (4.41) |
| ETD (95% CI) vs. PBO | | -4.5 (-12.1 to 3.7) | -5.8 (-13.4 to 2.4) | -1.3 (-9.2 to 7.3) | | -25.0 (-37.0 to -10.7)** | -14.8 (-28.6 to 1.7) |
| UACR ≥30 mg/g | -56.0 (7.48) | -74.3 (4.61) | -78.1 (3.50) | -71.2 (4.38) | -38.6 (9.37) | -69.7 (3.83) | -75.1 (3.23) |
| ETD (95% CI) vs. PBO | | -41.6 (-64.2 to -4.7)* | -50.3 (-68.8 to -20.9)** | -34.5 (-58.5 to 3.4) | | -50.7 (-66.6 to -27.1)*** | -59.5 (-72.7 to -39.8)*** |

Data are estimate (SE) unless stated otherwise. *$P < 0.05$, **$P < 0.01$, ***$P < 0.001$ vs. PBO from an MMRM model for post-baseline measures: log(actual measurement/baseline) = log(baseline value) + country + sex + prediabetes status at randomization (for SURMOUNT-1) or type of AHM used at randomization (for SURMOUNT-2) + treatment + time + treatment*time (type III sum of squares).
AHM, anti-hyperglycemic medication; CI, confidence interval; ETD, estimated treatment difference; MMRM, mixed model for repeated measures; PBO, placebo; SE, standard error; TZP, tirzepatide; UACR, urine albumin-to-creatinine ratio.

**Supplemental Table 4. Change from baseline at week 72 in non-indexed eGFR for SURMOUNT-1 and SURMOUNT-2**

| | SURMOUNT-1 | | | SURMOUNT-2 | | |
|---|---|---|---|---|---|---|
| Parameter | PBO (*N*=643) | Pooled TZP 5 mg, 10 mg, and 15 mg (*N*=1896) | LSM Change Difference (95% CI) vs. PBO | PBO (*N*=315) | Pooled TZP 10 mg and 15 mg (*N*=623) | LSM Change Difference (95% CI) vs. PBO |
| Cr-eGFR, ml/min per 1.73 $m^2$ | -0.4 (0.57) | -10.4 (0.32) | -10.0 (-11.2 to -8.7)*** | -3.4 (0.83) | -9.6 (0.50) | -6.2 (-8.1 to -4.3)*** |
| Cys-C-eGFR, ml/min per 1.73 $m^2$ | -1.4 (0.61) | -7.2 (0.33) | -5.8 (-7.2 to -4.5)*** | 2.4 (0.98) | -2.4 (0.58) | -4.8 (-7.0 to -2.6)*** |
| Cr-Cys-C-eGFR, ml/min per 1.73 $m^2$ | -0.8 (0.54) | -8.6 (0.30) | -7.8 (-9.0 to -6.6)*** | 0.4 (0.88) | -5.3 (0.52) | -5.8 (-7.8 to -3.8)*** |

Data are LSM (SE). ****P* < 0.001 vs. PBO from MMRM model for post-baseline measures with baseline value, analysis country, sex, prediabetes status at randomization (SURMOUNT-1) or type of AHM used at randomization (SURMOUNT-2), treatment, time, treatment*time (type III sum of squares).
AHM, antihyperglycemic medication; CI, confidence interval; Cr-Cys-C-eGFR, creatinine-cystatin-C-based estimated glomerular filtration rate; Cr-eGFR, creatinine-based estimated glomerular filtration rate; Cys-C-eGFR, cystatin-C-based estimated glomerular filtration rate; LSM, least squares mean; PBO, placebo; SE, standard error; TZP, tirzepatide.
